# Australian families living with rare disease: experiences of diagnosis, health services use and needs for psychosocial support

**DOI:** 10.1186/1750-1172-8-22

**Published:** 2013-02-11

**Authors:** Matilda Anderson, Elizabeth J Elliott, Yvonne A Zurynski

**Affiliations:** 1 Australian Paediatric Surveillance Unit, Kids Research Institute, Westmead, NSW, 2145, Australia; 2 Discipline of Paediatrics and Child Health, Sydney Medical School, The University of Sydney, Sydney, Australia; 3 The Sydney Children’s Hospitals Network, NSW, Australia

**Keywords:** Rare disease, Child, Disease burden, Health services

## Abstract

**Background:**

Families of children living with a rare disease report significant health and social burden, however, few studies have systematically examined family needs by using validated tools to assess the scope and extent of this burden. Our aim was to develop a comprehensive survey to assess health, psychosocial and financial impacts on Australian families caring for a child with a rare disease.

**Methods:**

We developed a self-administered survey for parents/carers incorporating pre-validated tools. The survey included questions about experiences of diagnosis, health services use and needs, needs for peer and financial supports. Forty-seven families attending the state-wide Genetic Metabolic Disorders Service at the Children’s Hospital at Westmead, Sydney were invited to participate.

**Results:**

Of 46 families who received the survey, 30 (65%) completed it. Most (93%) found the survey acceptable and relevant (91%). Patients were 1–17 years old, 14 (47%) male, and 12 (40%) non-Caucasian. Eighteen (60%) had a lysosomal storage disease and 12(40%) had a mitochondrial disorder. Eleven (38%) saw 3–5 doctors and four (14%) saw 6–10 doctors before receiving the correct diagnosis; 43% felt diagnosis was delayed. Four (13%) were dissatisfied with the way diagnosis was given, due to insensitive style of communication, inadequate information and psychological support. Psychosocial impact was moderate to high for 90% of families and the level of impact was not dependent on the level of health functioning of the child. Twenty-six (87%) wanted, but only 13(43%) received, information about peer-support groups. The 30 children accounted for 168 visits to general practitioners and 260 visits to specialist doctors; 21 (70%) children had at least one admission to hospital, including one who had 16 admissions in the previous 12 months. Most families (77%) received financial assistance but 52% believed this was insufficient. Families benefited from a specialised multi-disciplinary clinic but called for patient-held electronic medical records.

**Conclusions:**

Australian families caring for children with genetic metabolic disorders are adversely impacted by delays in diagnosis, lack of easy access to peer support groups and lack of psychological support. Further research is needed to estimate economic impact and to analyse health service delivery models for children with rare diseases in Australia.

## Background

Families living with rare diseases have traditionally received little attention from health authorities, clinicians and researchers. Rare diseases are difficult to diagnose and treat and there is often a lack of appropriate health services, skilled health professionals and effective treatment options [1]. Many rare diseases are chronic and complex and associated with physical, intellectual or neurological disabilities. As a result, the psychosocial and emotional impacts are significant for patients and families, and often compounded by a lack of appropriate peer and community support services [2]. These impacts on families have not been systematically explored or recorded in Australia [1,3]. An evidence base is needed to support the development of health and social policy, new health service models and peer-support groups and to underpin a coordinated National Plan for Rare Diseases [3].

The definition of a rare disease varies but in Europe it is accepted as any disease with a population prevalence of <1/2000 [2,4]. There are between 6000 and 8000 rare genetic diseases, in addition to rare non-genetic conditions, and when combined they affect approximately 6-10% of the population [2]. Extrapolating to the Australian population of 22.5 million, this equates to up to 2.2 million Australians, including up to 400,000 children [5].

The impacts of rare diseases on patients and families have been studied in Europe, United Kingdom and in the USA. Such studies have often been led by national organisations representing patients living with a rare disease, including the National Organization for Rare Disorders (NORD), Rare Diseases UK (RDUK) and the European Organisation for Rare Diseases (EURORDIS) [6-8]. These surveys highlighted diagnostic delays, many patients experiencing rejection by health professionals, and significant financial and social impacts on families [8]. However, few of these surveys have used psychometrically validated tools. Smaller studies have been conducted in Australia to investigate the burden of rare disease, but these have been limited to single disease groups [9,10]. Studies abroad have highlighted the additional difficulties that can be experienced by children and adolescents suffering from a rare or chronic disease, including problems with school functioning, self esteem and social interaction [11]. Australia requires wide-ranging data on the impacts of rare diseases using a survey appropriate for the Australian health care setting to inform the development of improved health care and support services for young patients and their families.

Our aim was to develop a comprehensive survey that includes validated patient-reported outcome tools appropriate for the Australian setting, which would enable systematic assessment of the impacts of rare diseases on children and their families and could be applied across broad disease groups. We describe the development of a new survey and the outcomes of a pilot study in Australian families who have a child with a genetic metabolic disorder.

## Methods

### Development of the survey

We used the World Health Organisation’s framework for International Classification of Functioning and Health (ICF) to support the development of a new survey tool to identify and investigate important topics for patients and families with rare diseases [12]. The ICF provides a broad multifactorial, multidisciplinary framework reflecting the diverse and interrelated determinants of health, and can be applied to the study of any chronic condition including rare diseases [12]. From the existing literature, we identified key areas for inclusion in our survey, which were both significant for patients and families as well as important for health authorities and service provision planning. These were broadly categorised into the following sections:

• Demographics

• Experience of diagnosis

• Health related function of the patient

• Impact on the family

• Use of health services

• Use of support services

A literature review was performed to identify existing questionnaires and psychometrically validated tools that could be included in the new survey and would fit our goals and outcomes in accordance with patient-reported outcome assessment use models [13]. We selected tools that were:

• relevant to a paediatric population

• generic (not diseases-specific)

• self-administered either in paper or on-line formats

• simple for parents to complete

• easy to score and analyse.

Interview-based tools were automatically excluded. Three validated and suitable tools were identified: the Health Utilities Index Mark II (HUI- II) (assessing health functioning) [14]; the Impact on Family scale (IOF) [15]; and the Royal Alexandra Hospital for Children Measure of Function (RAHC MOF) [16]. The HUI-II is validated for children aged 5 years or more while the RAHC MOF is validated for all paediatric/adolescent age groups [14,16]. The RAHC MOF was developed in an Australian paediatric hospital as a global measure of health and is designed to be completed by either a medical professional or parent to categorise health functioning of the child. A score of one (superior functioning) to ten (need for constant supervision, high medical dependency) is then allocated (Table 1) [16]. The IOF scale is a 24-item tool using a 4-point Likert scale (strongly agree to strongly disagree) to score the level of agreement with each of the 24 statements. Each item in the IOF has been tested for reliability and psychometric validity [15]. The total score for the IOF is based on 15 out of the 24 items and ranges from 15 (low impact on the family), to 60 (very significant impact on the family), while a score of 30 indicates moderate impact [15]. The HUI-II, the RAHC MOF and the IOF were included in our final survey in their entirety to preserve the psychometric validity and to enable interpretation of scores for each of these instruments.

**Table 1:** **Categories and descriptions of the RAHC MOF scale**[16]

**Category**	**Description**
1. Superior	No symptoms; physically able; excellent relationships with family and friends; wide range of extra-curricular activities; doing well at school/preschool; developing normally; everyday problems never get out of hand.
2. Good in all areas	Virtually no symptoms; usually copes well; physically able; good relationships; normal play & leisure activities; school/preschool OK; may have problems when stressed but these are short lived and only occasionally get out of hand.
3. No more than slight problems	Some significant symptoms, only briefly get out of hand; sometimes child gets distressed; short term or little interference with mobility or relationships or play & leisure activities; school/preschool may be slightly affected or affected for a short time.
4. Some difficulty in a single area but generally pretty well	Mild symptoms which recover quickly with treatment; any distress or disability does not stop child from doing most things at that age; some anxiety or irritability or brief mood changes; minor effect on mobility or school/preschool or relationships or play & leisure activities; problems may persist but may only be recognized by those who know the child.
5. Variable problems in some but not all areas	Moderate symptoms have significant disabling effect on child; minor to moderate effect on mobility; school/preschool may be affected; may need special education; in some situations may seem O.K.; mainly managed in outpatient clinic or family doctor.
6. Severe problems in one area OR moderate problems in most areas	Severe symptoms having a major effect on child’s life; restricted mobility; relationships or play & leisure activities are affected; child is distressed or has difficult behavior; some relationships are maintained; learning difficulties or problems with or missing school; likely to have been seen by specialist.
7. Major problems in several areas AND unable to function in one of these areas.	Severe, almost constant symptoms; child is distressed, withdrawn or has strange or aggressive behaviour; significant limitations on mobility or school/preschool or relationships or play & leisure activities; specialist management needed.
8. Unable to function in almost all areas	Very severe symptoms; child is very distressed; likely to be confined to bed; unable to go to school/preschool; may be in hospital but child is not entirely dependent on others.
9. Needs nursing supervision	Confined to bed; in hospital; very severe symptoms but stable; needs help with self-care which a child the same age can do without help.
10. Needs constant supervision	High (24 hrs) medical dependence e.g. In intensive care unit; life-threatening symptoms.

The EurordisCare surveys [2,8] and the Australian Rett Syndrome survey [10] were also assessed and questions on economic impacts, experiences of diagnosis, and use of support groups were adapted from these surveys and included in ours. A small number of additional questions about the use of and need for health services, participation in research and need for peer support groups were constructed by us, using multi-item Likert scale answer formats. These additional questions were simple and asked for factual information (eg. number of visits to specialists in the last 12 months) or for opinion (eg. How interested are you in finding and utilising support groups and organisations?). The additional questions were not psychometrically tested. The first draft of our survey was reviewed by researchers and clinicians working in the field of rare diseases. The subsequent draft was reviewed by 5 lay people and feedback on question structure and clarity was incorporated into the final version.

The final survey was 10 pages long and contained 7 sections covering demographics, experiences of diagnosis, health related functioning of the child, use of and needs for health services, impact on family, and use of and need for financial and social support services. Health services use was categorised into hospital admissions (defined as a stay in hospital of ≥8 hours), visits to outpatient clinics and visits to the emergency department. The survey was produced in hard copy and also formatted for on-line completion using the LimeSurvey program [17].

We piloted the survey by inviting 47 families currently engaged with the Genetic Metabolic Disorders Service at The Children’s Hospital at Westmead, in Sydney, Australia to participate. This state-wide clinic manages a diverse range of metabolic disorders, including children with mitochondrial and lysosomal storage disorders. Children with these conditions were chosen for the pilot study as they represent a range of phenotypes and a broad spectrum of health status and functioning.

The 47 families were sent a letter of invitation, an instruction sheet, the survey, a reply-paid envelope and a brief evaluation form asking about the acceptability of the survey, including its length and content. Families were asked to complete the questionnaire either on-line or to return the completed paper version by reply-paid post. A reminder letter was sent after two weeks. Data from both the paper and online versions were entered onto the Statistical Package for the Social Sciences (SPSS) and analysed using descriptive statistics. Ethical approval was granted by the Children’s Hospital at Westmead Human Research Ethics Committee (10/CHW/75).

## Results

### Sample characteristics

One of the 47 families had changed address and did not receive the survey. Of the 46 families who received the survey, 30 (65%) returned the survey. Only two chose to complete the survey on-line. In the majority (80%) the child’s mother completed the survey. Among the children, approximately half were male, most were less than 10 years of age and 12(40%) identified with an ethnicity other than Caucasian (Table 1). Eighteen (60%) children had a lysosomal storage disorder including one of the Mucopolysaccharidoses (n = 12), Fabry Disease (n = 5), and Pompe Disease (n = 1). Twelve (40%) children had a mitochondrial disorder, including mitochondrial respiratory chain disorders with various defects (n = 5), Mitochondrial Encephalopathy with Lactic Acidosis and Stroke-like episodes (MELAS) (n = 3), Kearns-Sayre syndrome (n = 2), Pearson Syndrome (n = 1) and Leigh syndrome (n = 1) (Table 1).

Twenty-five (83%) of the children had siblings and 8 of the siblings had the same disorder including 5 siblings who had died from the disorder. The other three siblings who had the same disorder were older than 18 years and no longer attached to the Genetic Metabolic Disorders Service at the Children’s Hospital at Westmead (Table 2).

**Table 2:** Characteristics of 30 patients with genetic metabolic disorders

**Patient characteristics**	**N(%)**
**Gender**	
Male	14(47)
Female	16(53)
**Age groups**	
0-5 years	8(27)
6-10 years	11(37)
11-15 years	7(23)
>15 years	4(13)
**Diagnosis**	
Lysosomal Storage Disease	18(60)
Mitochondrial disease	12(40)
**Ethnicity**	
Caucasian	17(57)
Asian	5(17)
Middle Eastern	5(17)
Other	2( 7)
**Country of Birth**	
Australia	28(93)
Malaysia	2( 7)
Has at least one sibling	25(83)
Other family members affected by the same rare disease	12(40)

### Experiences of diagnosis

Only one child, whose parent had been diagnosed with the same disorder, was diagnosed before birth. Two (7%) children were diagnosed at birth, 9(30%) before 12 months of age, 9(30%) between 12 months and 3 years of age, and 9(30%) at 4 years of age or more. The majority (73%) of families reported that symptoms or signs of the disease began before the age of 4 years. Eleven (38%) children had seen 3–5 doctors and 4 (14%) children had seen 6–10 doctors before receiving the correct diagnosis. Five families (17%) reported that their child had initially been given a wrong diagnosis. Twelve (43%) believed their child’s diagnosis could have been made earlier, citing reasons such as lack of knowledge by health professionals about the disease, and unavailable or delayed testing.

Twenty-three (77%) families were ‘very satisfied’ or ‘satisfied’ with the way in which they were informed of their child’s diagnosis, while 3 families were neither satisfied not dissatisfied. The 4(13%) families who were dissatisfied with the way the diagnosis was given cited reasons such as an insensitive style of communication, no offer of support or counselling, and inadequate provision of information about the disease (Table 3).

**Table 3:** Examples of comments about the way the diagnosis was given to families

**Positive comments**	**Negative comments**
‘*The doctor was very assuring and helpful*…*offered a counsellor and a number to call if I needed any information no matter how important*’	‘*I was on my own and I was offered no support or counselling*… *I was told with what seemed to be no empathy or sensitivity*’
‘*The doctor was honest and did not find it easy to give such bad news*’	‘*Too many people in the room*’
‘*The haemotologist met with me and my husband alone and invited the geneticist along as well*. *They also followed with a letter and some fact sheets explaining the disease*. *This helped because it was hard to take it all in*.’	‘*The paediatrician walked out half*-*way through the diagnosis*’
‘*Very satisfied because everyone involved knew what they were saying and doing*’	‘*Paediatric ophthalmologist not very sensitive*; *told us loudly with their back to our daughter*… *who is old enough to understand*. *Then referred to the* [*Genetic*] *metabolic clinic at Westmead*….’
‘*The staff in the genetic clinic were very informative and my husband and I could ask questions*’	

### Health-related function

The median score on the RAHC MOF was 4 (some difficulty in a single area). Eleven (37%) children scored 1 or 2 (superior or good in all areas), 3 (10%) scored 3 or 4 (slight problems or some difficulties), 12 (40%) scored 5 or 6 (variable problems in some areas or severe problems in at least one area), and 4(13%) scored 7 or 8 (major problems or unable to function in almost all areas). No child scored 9 or 10 (needs nursing supervision).

One child had a cochlear implant, two used hearing aids, five had extremely poor mobility (‘unable to control or use arms or legs’) and eight used a wheelchair. Families with children aged over 5 years (22 of the total 30) were asked more detailed questions about health functioning. Fifteen (68%) of the 22 had pain or discomfort, and one child had severe pain not relieved by medications. Eight (36%) reported impaired abilities in speech, hearing and communication.

### Use of health services

The level of health service use varied among the 30 families. Three families reported visiting hospital more than 50 times over the previous 12 months (for clinics, diagnostic or treatment procedures or to attend the emergency department) although nine families had not visited hospital at all in the past year. Admissions to hospital in the previous 12 months ranged from none for 10(33%) families, to 16 for one child. Sixteen (53%) children reported 1–3 admissions and four (13%) reported 4 or more admissions.

Families reported using the services of multiple doctors. General practitioners (GPs) were accessed most often. Twenty-four children (80%) had visited their GP in the previous 12 months accounting for a total of 168 visits, an average of seven visits per child per year (Table 4). Frequently accessed specialists included paediatricians, geneticists, eye specialists, dentists, neurologists and surgeons (Table 4). All 30 children had visited at least one specialist, including a dentist, accounting for 260 visits over the previous 12 months, an average of 8 visits per child per year. Use of allied health professionals including speech therapists, physiotherapists and occupational therapists was also high: 24 children visited an allied health professional at least once, accounting for 268 visits in the previous 12 months, an average of 11 visits per child per year (Table 4).

**Table 4:** Visits to medical practitioners and allied health professionals by the 30 families in the last 12 months

**Medical practitioner**	**Number of patients visiting at least once**	**Total number of visits for the whole group**
**General Practitoner**	**24**	**168**
**Specialists**	**30**	**260**
Paediatrician	18	69
Geneticist	16	41
Eye Specialist	24	34
Cardiologist	16	30
Dentist	17	28
Neurologist	11	20
Surgeon	13	20
Pain Specialist	4	4
Other Specialist	12	14
**Allied Health Professionals**	**24**	**268**
Speech Pathologist	7	70
Physiotherapist	10	63
Occupational Therapists	12	58
Social Worker	6	33
Dietician	10	28
Genetic Counselor	4	6
Other Allied Health Professional	3	10

Most (80%) families felt they had adequate access to health services. Thirteen (43%) identified barriers to accessing services including: distance to travel, need for sibling care, cost, lack of available services, time lost from work and lack of referral. Twelve (40%) of the families felt that there were no barriers to accessing health services. Four commented on the long waiting times to see specialist medical practitioners and other health professionals.

When asked about the role of the GP in their child’s care, the majority (73%) of families agreed the GP’s role should be to coordinate services and 73% also agreed that their GP had adequate knowledge of their child’s disease. Two-thirds (60%) indicated that they had a health professional who coordinated all aspects of health care for their child. Twenty-three (77%) agreed that accessing health services through a specialised centre housing coordinated multidisciplinary services (namely the Genetic Metabolic Disorders Service) improved their experiences in accessing health care. The great majority (93%) indicated that an electronic health record containing all relevant medical history would improve their experiences of accessing health care.

Although our survey did not include any specific questions about access to treatment, four families raised issues around equitable access to treatments, indicating a perceived lack of access to drugs used overseas but not approved in Australia.

### Impact on family

**Figure 1:**
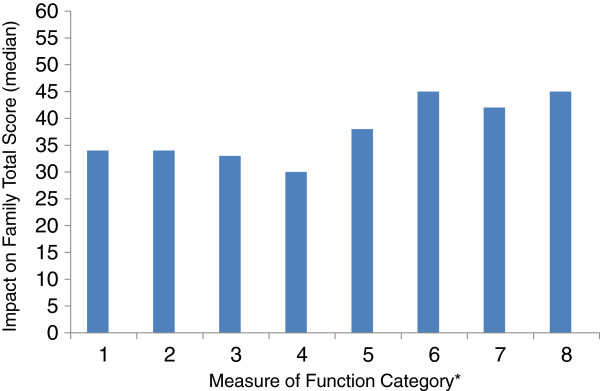
Distribution of the Impact on Family Score by MOF* level of function scores. * MOF Score definitions: 1- Superior functioning. 2- Good in all areas. 3- No more than slight problems. 4- Some difficulty in a single area. 5- Variable problems in some areas. 6- Severe problems in one area or moderate problems in most areas. 7- Major problems in several areas. 8- Unable to function in most areas. 9- Needs nursing supervision. 10- Needs constant supervision.

The median IOF score was 36.5 suggesting a moderate level of impact of disease on families, however the scores ranged from 19 to 56 suggesting a very wide range of impacts. Only 3 (10%) families scored < 30 on the IOF (indicating a low level of impact) while 21(70%) scored between 30 and 45 (indicating significant impact) and 6 families scored 46 or more (indicating very serious impact). We analysed the relationship between the health function score (RAHC MOF) and the IOF and found that even families whose children scored 1 or 2 on the (RAHC MOF) and had superior or good health function scored over 30 on the IOF, indicating at least moderate impact on the family (Figure 1).

Most families (77%) were receiving some form of financial support through government programs such as the Carer Allowance [18]. However, about half (52%) indicated that the financial assistance they received was inadequate to cover their needs and 22(73%) said that additional income was needed to cover medical expenses (IOF question 1).

Seventy percent of families indicated that they had become closer because of shared experiences when living with a child with a rare disease (IOF question 21) and 77% had positive experiences with relatives who were “understanding and helpful” (IOF question 24).

On a scale of 1 (not stressed most of the time) to 5 (highly stressed most of the time) 23 (76%) families scored 3 or more and 13(43%) scored 4 or 5, suggesting a significant level of stress. Seventy per cent of families felt that their child’s illness resulted in the family seeing friends and relatives less often than desired (IOF question 3) and 77% said it was difficult to find a reliable person to care for their child (IOF Question 7). Seven respondents were receiving support from a social worker, counsellor, psychologist, or psychiatrist.

### Support services and information needs

Over a half (57%) of families were ‘interested’ or ‘very interested’ in being involved with a peer support group but less than half had found a relevant group in Australia and 37% had looked for a support group overseas (Table 5). Information regarding relevant support groups was provided to 43% of families at diagnosis, but 87% thought this information should be routinely offered at the time of diagnosis. Families were interested in keeping abreast of latest research (90%) and 73% were prepared for their child to participate in relevant research studies.

**Table 5:** Reported needs for information about support groups, diseases and research

**Question**	**Number of responses**	**Yes**	**No**	**Don’t Know**
		**N (%)**	**N (%)**	**N(%)**
**Need for Support Groups**				
Would you like information about support groups at diagnosis?	28	26 (87%)	1 (3%)	1 (3%)
Was support group information provided at diagnosis?	30	13 (43%)	13 (43%)	4 (13%)
Are you interested in being involved with support groups?	29	17 (57%)	10 (33%)	2 (7%)
Have you searched for support groups overseas?	30	11 (37%)	19 (63%)	0
Have you found a support group in Australia specific to your child’s rare disease?	29	13 (43%)	13 (43%)	3 (10%)
**Access to information and research**				
Do you believe you have been provided with adequate information about your child’s disease?	30	23 (77%)	5 (17%)	2 (7%)
Have you been provided with adequate information about you and your child’s legal and social rights?	29	11 (37%)	12 (40%)	6 (20%)
Have you been provided with adequate information about financial assistance?	29	13 (43%)	13 (43%)	3 (10%)
Are you interested in being kept informed of current research and clinical trials related to your child’s disease?	30	27 (90%)	2 (7%)	1 (3%)
Are you interested in your child participating in relevant research studies?	29	22 (73%)	3 (10%)	4 (13%)

### Relevance and acceptability of the survey

All 30 families completed the survey evaluation. Twenty-nine (97%) indicated that the survey questions were easy to understand and 28 (93%) felt that the length of the survey was acceptable. Most (83%) families thought the questions were relevant to their experiences and none indicated that they would like any questions left out. Six (20%) suggested that some important areas had not been covered including impacts on siblings and the reactions of the general community to their child. Most (26, 87%) took less than one hour to complete the survey, including the 7(23%) who spoke a language other than English at home.

## Discussion

Data on the experiences of patients living with a rare disease are scarce in Australia. Our study addresses this gap and demonstrates impacts on families and on health services in a small, well described group of Australian children diagnosed with a genetic metabolic disorder and attending a state-wide multidisciplinary clinic in Sydney, New South Wales. To assess impact we developed a comprehensive survey, which was judged acceptable and relevant by families living with a child affected by a rare genetic metabolic disease. Our survey is generic and could be applied across a spectrum of rare diseases, given the phenotypically diverse range of metabolic diseases represented by our sample. We propose to study the relevance of this survey to a wider range of rare diseases in the future. Our survey is the first to use validated tools to assess impact on families and to correlate this with health functioning in the child. Such data are needed to inform future development of health and support services for families.

This study has some limitations. Our sample was small, was sourced from a specialised clinic in a tertiary paediatric hospital and included only selected rare disease groups, thus the findings cannot necessarily be extrapolated to other patient groups. Nevertheless, the cohort included 65% of all children with lysosomal storage diseases and mitochondrial diseases from a state-wide service, where no other such service exists, and is therefore likely to be representative of families living with these conditions in NSW and probably throughout Australia. Our response rate (65%), was high compared with the EurordisCare2 survey (33%) [8], and EurordisCare3 survey (30%) [2]. The RDUK survey did not report a response rate [7].

Although most children in our study were born in Australia and over half were Caucasian, Asian families (17%) and Middle Eastern families (17%) were over-represented compared with the reported proportion of these groups within the general Australian population (7% Asian and 1.4% North African and Middle eastern) [19], indicating a high burden for these communities, and a need for awareness raising about genetic disorders. Often the child attending the clinic was not the only affected child in the family. Eight families reported a sibling who was currently affected by the same disorder including five families who had one or more children who had died due to the rare disorder, highlighting the significant and enduring burden of genetic diseases. Such families may benefit from improved access to genetic counselling services.

Forty per cent of families believed that their child’s diagnosis could have been made earlier and many saw multiple doctors before receiving the correct diagnosis. These findings echo results from the EurordisCare2 surveys and the RDUK survey [2,7,8]. Delay in diagnosis can have medical consequences such as delayed treatment, unnecessary tests, and psychological stress for the family. Our findings indicate a need for better education of health professionals during undergraduate and post-graduate studies, better awareness of existing information resources and development of new resources to support clinical care. Such resources could include clinical guidelines on diagnosis and treatment, educational modules which could be delivered via the internet and lists of specialised clinics and referral pathways.

The way in which diagnosis was given was satisfactory for most families and only four were ‘not satisfied’ or ‘very unsatisfied’. This compares favourably to results from the EurordisCare2 survey, in which 35% of respondents indicated that they were dissatisfied with the way diagnosis was given [8]. Our sample, however, was very small and included only families who were already attached to a multidisciplinary genetic metabolic service. Families who had negative experiences of receiving the diagnosis may have received the diagnosis from other health professionals outside of the Genetic Metabolic Disorders Service and before being referred to the state-wide service. Few families in our study were offered psychological support or counselling at the time of diagnosis, despite feeling ‘*devastated*’, ‘*confused*’, ‘*heart*-*broken*’ and ‘*in shock*’ when given the diagnosis. This highlights the need for routine psychological support following diagnosis.

The level of health related functioning among children with a rare disease can vary widely, however impacts on mobility, cognitive function, emotional functioning and day-to-day activities are common [20]. Caring for a child with a rare condition has a high impact on families even when the child has relatively mild disease. In our study, families of children who scored in the “good health function” range of the (RAHC MOF), scored in the moderate level of family impact on the IOF, indicating that there are factors apart from the level of health function of the child which contribute to the impact on family. Indeed, an Australian study on stress in mothers and fathers of children with fragile X syndrome found the strongest predictors in these families were marital satisfaction (for mothers) and the child’s adaptive abilities (for fathers) [10]. Caring for children with a rare disease has been linked with significant stressors such as the need to accept the diagnosis and adaptation to new roles, increased demands on time, and requirement to manage the child’s day-to-day care [21]. In our study, frequent burdens for families included little time to see relatives and friends and difficulties finding a reliable person to care for their child. High levels of psychological and financial stress were reported by over 75%.

The high levels of reported stress suggest an unmet need for psychological support from mental health professionals, counsellors or peer support groups, however, only 7 of the 30 families were receiving support from a mental health professional and less than half had found a relevant peer support group. Over a third (37%) had searched for a support group overseas, suggesting a lack of availability or access to support groups in Australia. The importance of support groups is being increasingly recognised, through their ability to connect to patients and families via the internet, as well as collaborating with research groups [22]. Organisations such as the Association of Genetic Support of Australasia (http://www.agsa-geneticsupport.org.au) and Genetic Alliance groups across Australia act to promote peer support, however peer support organisations are often poorly resourced and lack coordination. There is also lack of a coordinated approach to advocacy for people living with rare diseases in Australia. A newly established organisation, Rare Voices Australia (http://www.rarevoices.org.au) aims to facilitate better access to and coordination of peer support services and to advocate at the national level for people living with a rare disease.

Our results illustrate the frequent use of health services for some children with rare diseases, including 16 admissions for one child and >50 visits to hospital for three children in a 12 month period. Given such frequent use of health services, the finding that 80% of families felt they had adequate access to health services was positive. However, participants in our study were recruited via a specialised multidisciplinary service and we therefore anticipated high levels of satisfaction with the provision of health services. Nevertheless, families identified key difficulties in accessing care, including practicalities such as time for travel to clinic and long waiting times to see specialist doctors and allied health professionals. The latter suggests a substantial burden for clinics with limited resources and staff. Service delivery via appropriately resourced specialised centres housing medical practitioners, allied health staff, pathology, pharmacy and access to equipment and information in one location and patient held electronic health records might further improve their experiences when accessing many different health professionals. Our survey did not specifically ask whether there were delays to see staff in the multidisciplinary Genetic Metabolic Service or outside of the service, and this will be taken into consideration when further developing our survey.

We were interested in the role of the general practitioner in the care of children with rare diseases and complex and highly specialised needs. An article published in the Medical Journal of Australia by two GPs highlighted the lack of a defined role for the GP when dealing with patients with a rare disease and their families, as well as a lack of available support and resources for GPs [23]. GPs are in the front line of the health system and can empower and advocate for patients living with a rare disease by coordinating care and making decisions about appropriate referrals for diagnosis and treatment as well as providing routine general and preventative health care such as vaccinations. The majority of families (73%) thought their GP was in a position to coordinate the wide range of services their child required. GPs have called for a systematic, primary-care approach to rare disease to assist them in managing patients and families, thereby reducing diagnostic delays, providing care coordination, and providing an extra avenue for access to psychological support [23].

## Conclusions

Results from this pilot study of 30 Australian families living with a rare metabolic disease indicate that these children have significant disability and health needs and that families are emotionally and financially stressed. Most suggested that information about the disease, details about support groups and psychological support for the family should be routinely offered at the time of diagnosis. Although all families were accessing health care via a multidisciplinary clinic they felt that their experiences could be improved with better coordination of care and introduction of electronic health records which could be accessed by the many different health professionals with whom they interact. Organisations such as EURORDIS and NORD, play an important role in advocating for patients with rare diseases. We believe that Australian families living with rare disease would benefit from an overarching national organisation to coordinate advocacy for rare disease strategy and policy in Australia to improve access to health care and financial and community support. We have begun a large study using a revised version of our survey and involving approximately 500 children living with a variety of rare diseases, to further inform patient needs.

## Competing interests

Matilda Anderson has no competing interests.

Elizabeth Elliott has no competing interests.

Yvonne Zurynski has no competing interests.

## Authors’ contributions

YZ conceived the study, identified relevant literature, drafted the first version of the survey, developed the survey methods, supervised MA, analyzed and interpreted results and significantly contributed to writing and editing of the manuscript. MA performed a literature search, developed the survey, performed data entry and analysis, interpreted results and significantly contributed to writing of the manuscript. EE jointly conceived the study, provided expert advice on survey development and survey methods, supervised MA, and provided expert comment on the manuscript. All authors read and approved the final manuscript.
